# Oral Administration of the Probiotic *Lactobacillus casei* Ameliorates Gut Morphology and Physiology in Malnourished-*Giardia intestinalis*-Infected BALB/c Mice

**DOI:** 10.5402/2013/762638

**Published:** 2013-09-21

**Authors:** Geeta Shukla, Sumedha Singh, Angela Verma

**Affiliations:** Department of Microbiology, Basic Medical Sciences, Panjab University, Chandigarh 160014, India

## Abstract

Malnutrition reduces the host immunity and enhances the host susceptibility to various diseases. The present study describes the effect of oral administration of probiotic *Lactobacillus casei* to malnourished-*Giardia*-infected BALB/c mice with respect to surface alterations and brush border membrane enzyme activity of the small intestine. It was observed that probiotic feeding either prior to or simultaneously with *Giardia* infection to malnourished mice led to significantly enhanced activity of disaccharidases compared with malnourished and *Giardia*-infected mice. Scanning electron microscopy also revealed less mucosal damage in the villi of small intestine of probiotic-fed malnourished-*Giardia*-infected mice compared with completely damaged, mummified, or blunted villi of malnourished-*Giardia*-infected mice. The findings indicate that probiotics can be used as the prophylactic candidate in abrogating the gut and intestinal dissacharidases anamolies in malnourished hosts suffering from the intestinal diseases.

## 1. Introduction

Malnutrition and infections are quiet complimentary and show combined effects that worsen the health and continue to be an important cause of morbidity and stunting of growth among children in developing countries [1, 2]. It is well documented that more than 90% of the world's stunted children live in Africa and Asia, where rates of stunting are 40% and 36%, respectively, [3]. In India, 50% of child deaths are due to malnutrition while 46% of children under five years of age in rural India and 33% in urban India are underweight, with 16% being severely undernourished and 48% stunted. 

Malnourished individuals are more susceptible to various diseases such as typhoid, malaria, measles, giardiasis, and pneumonia [1, 4]. *Giardia intestinalis* is among the major health concerns observed in economically disadvantaged populations of developing countries. Other than in malnourished individuals, giardiasis is more common in young children as well as in homosexual, immunocompromised people with the highest prevalence of 20%–30% percent in developing countries [5, 6]. The parasite mainly inhabits the small intestine of humans and symptoms include flatulence, bloating, constipation, abdominal cramps, diarrhea, malabsorption, and growth retardation. 

Nutritional interventions are considered as an important approach to reduce the morbidity and mortality from gastrointestinal diseases in malnourished individuals [7]. Among various nutrient interventions, probiotics (particularly lactic acid bacteria) are generally regarded as safe for their inhibitory and immunomodulatory activities, are much in consideration, and are defined as live microorganisms thought to be beneficial, safe, effective, and cheap to the host [8]. 

Various clinical and experimental studies have shown that giardiasis induces both morphological and biochemical changes in the intestine mainly with respect to marked decrease in transport of nutrients and activity of the brush border enzymes [9–11]. Khanna et al. [12] have also shown a direct correlation between giardiasis and brush border membrane damage, in immunocompromised mice, while Prentice et al. [13] have documented altered permeability and prolonged intestinal injury in malnourished children [12, 13]. Earlier, we have observed that the probiotic *Lactobacillus casei *supplementation even to malnourished/renourished mice has been effective in reducing the severity, duration, and pathological alteration in *Giardia-*infected mice. However, alterations in disaccharidases activities have not been studied and warrant further investigation [14, 15]. Thus, an attempt was made to assess the modulatory effect of probiotic *L. casei* particularly with respect to intestinal surface and brush border membrane enzyme alterations in the small intestine of malnourished-*Giardia intestinalis-*infected BALB/c mice.

## 2. Materials and Methods

### 2.1. Chemicals

All the chemicals for laboratory experiments were purchased from Sigma-Aldrich Corporation (Bangalore, India). De Mann Rogosa Sharpe (MRS) broth was obtained from Hi-Media Laboratory Pvt. Ltd. (Mumbai, India). 

### 2.2. Parasite

*Giardia intestinalis *(Portland Strain I) trophozoites were axenically grown in TYI-S-33 medium supplemented with antibiotic solution and pH was adjusted to 6.9 before sterilization with 0.22 *μ*m filter. Actively growing trophozoites (48–72 hours old) were harvested by centrifugation (2000 rpm for 15 minutes) after chilling in ice for 15 minutes and finally suspended in phosphate-buffered saline pH 7.2 (PBS) to contain 5 × 10^6^ trophozoites/0.1 mL [16].

### 2.3. Bacterial Strain

*Lactobacillus casei *MTCC 1423 was grown in MRS broth and maintained on MRS agar slants by regular subculturing at an interval of 15 days by being incubated at 37°C for 24 hours. For experimental inoculation, 18-hour-old culture of *L. casei* was sedimented by cold centrifugation at 8,000 rpm for 10 minutes and washed. Finally, pellet was resuspended in PBS to contain 1 × 10^10^ lactobacilli/mL and 0.1 mL was fed orally using blunt-ended feeding needle [16, 17].

### 2.4. Animals

Five-six-week-old BALB/c mice (18–20 grams) were procured from the Central Animal House, Panjab University, Chandigarh, India. The mice were housed under the standard conditions of light and dark cycle and were provided standard pellet diet (Hindustan Lever Products, Limited, Kolkata, India), or 4.3% protein pellet diet (Ashirwad Private Limited, Kharar Punjab, India) as per the groups and water *ad libitum.* Before supplementation to animals, water and feed were monitored for any parasitic contamination by Lugol's iodine, staining technique [16]. Stool samples of all the animals were examined for three consecutive days [18]. Only *Giardia*-free animals were employed for the study. Care and use of animals were in accordance with the guidelines of the institutional ethical committee.

### 2.5. Induction of Malnutrition

Normal mice were considered malnourished, when fed with 4.3% protein pellet diet up to 21 days. At the end of this period, animals which had lost about 35%–55% of their initial body mass were labeled as malnourished mice [14, 15, 19].

### 2.6. Groups of Animals

Animals were divided into seven groups and each group comprising of 24 mice. *Group I* (control): normal mice were fed orally with single dose of PBS (0.1 mL) daily for 30 days and were provided with standard protein pellet diet. *Group II* (malnourished): malnourished mice were fed orally with PBS (0.1 mL) daily and were given 4.3% protein pellet diet. *Group III* (*Giardia*-infected): normal mice were challenged orally with single dose of *Giardia *trophozoites (5 × 10^6^/0.1 mL) and were given standard protein pellet diet. *Group IV* (malnourished-*Giardia*): malnourished mice were challenged orally with single dose of *Giardia* trophozoites (5 × 10^6^/0.1 mL) and were given 4.3% protein pellet diet. *Group V* (malnourished-probiotic): these malnourished mice were fed orally with single dose of *L. casei *(1 × 10^9^ cfu/0.1 mL) daily and were provided 4.3% protein pellet diet. *Group VI* (malnourished-*Giardia*-probiotic): malnourished animals were challenged orally with single dose of *Giardia *trophozoites (5 × 10^6^/0.1 mL) and simultaneously with single dose of *L. casei *(1 × 10^9^ cfu/0.1 mL). These animals were provided 4.3% protein pellet diet and probiotic treatment was continued till the end of the experiment. *Group VII* (malnourished-probiotic-*Giardia*): malnourished mice were fed orally with single dose of *L. casei *(1 × 10^9^ cfu/0.1 mL) for 7 days and were provided 4.3% protein pellet diet. On 8th day these mice were simultaneously challenged orally with single dose of *Giardia *trophozoites (5 × 10^6^/0.1 mL) and probiotic (1 × 10^9^ cfu/0.1 mL). Thereafter, only probiotic treatment once a day was continued till the completion of the experiment. 

### 2.7. Enumeration of *Giardia *Cysts

After respective treatment, cyst count was monitored. Briefly, 0.1 gm of freshly passed faecal sample was mixed thoroughly with 1 mL formal saline using pestle motor. Cysts were stained with Lugol's Iodine and counted on every alternate day in the heamocytometer [15].

### 2.8. Followup of the Animals

Animals were sacrificed by cervical dislocation in batches of 6 on day 4 (establishment phase, 3–5 days), 9 (acute phase, 8–10 days), and 20 (decline phase, 19–21 days) postinfection (PI), respectively, for the estimation of small intestine mass, surface alteration of the small intestine, and intestinal enzymes.

### 2.9. Determination of the Small Intestine Mass

The entire small intestine was removed after sacrificing the animals by cervical dislocation and was weighed on electrical balance weight scale (SD-300, S.D fine chemicals Ltd., Chandigarh, India).

### 2.10. Preparation of Brush Border Membranes (BBMs)

Brush border membrane was isolated and purified from the small intestine as reported by Kessler et al. [20]. After sacrificing animals, the small intestine was removed, washed, and then homogenized in 5% (w/v) Tris Mannitol buffer (pH 7.2) and filtered through muslin cloth. An aliquot of the filtrate was used for protein and alkaline phosphatase analysis and labeled as crude BBM homogenate. BBM was further purified with addition of 10 mM CaCl_2_ to the filtrate with constant stirring at 37°C for 15 minutes and was cold centrifuged at 8000 ×g for 25 minutes. The supernatant was again cold centrifuged at 7000 ×g for 30 min. The pellet was resuspended in 2 mL of 50 mM sodium maleate buffer (pH 6.8) and labeled as purified BBM. Purity of the membrane was evaluated by comparing the alkaline phosphatase activities both in crude BBM and purified BBM preparation. 

### 2.11. Assay of Disaccharidases

Disaccharidases (sucrase, maltase, and lactase) in the BBM were assayed by measuring the D-glucose liberated from the respective sugars using the glucose oxidase peroxidase system of Dahlquist [21]. Substrates (sucrose, maltose, and lactose, 0.15 M) were prepared in 50 mM sodium maleate buffer, pH 6.8. Reaction mixture containing 0.1 mL of respective substrate and 0.3 mL sodium maleate buffer was taken in separate test tubes and was equilibrated by being kept at 37°C for 5 minutes. To each tube, 0.1 mL of sample was added and incubated at 37°C for 30 minutes for sucrase and maltase, 1 h for lactase. The amount of glucose liberated from respective sugars was estimated by the addition of 2.5 mL of glucose oxidase peroxidase reagent (Reckon GOPPAP kit, Vadodara, India) and kept at 37°C for 1 h. The optical density was measured at 500 nm. Enzyme activity was expressed as units/mg of protein, where one enzyme unit is defined as the amount of enzyme, which transformed 1 *μ*mole of the substrate per minute under standard assay conditions.

### 2.12. Alkaline Phosphatase Assay

Alkaline phosphatase in the intestinal homogenate was estimated by using p-nitrophenyl phosphate as substrate by the method of Bergmeyer [22]. Briefly, 0.5 mL buffered substrate (0.1 M Glycine-NaOH buffer with 5.5 mM p-nitrophenyl phosphate, pH 10.5) was added to the test tubes and kept at 37°C for 5 min for equilibration followed by the addition of 0.1 mL of sample (BBM). Reaction was stopped by adding 5 mL of 0.1 N NaOH. Absorbance was read at 420 nm and results were expressed as micromoles of p-nitrophenol formed per milligram of protein.

### 2.13. Scanning Electron Microscopy (SEM)

Surface alterations occurring in the small intestine were monitored by SEM. Mice from each group were sacrificed by cervical dislocation and upper portion of the small intestines was removed. The proximal part of the intestine was opened longitudinally, washed with PBS, stapled on cardboard, and fixed in 4% glutaraldehyde for 1 h. The samples were dehydrated in different grades of alcohol, that is, 50, 70, 80, 90, 100, and again 100% for 10, 15, 15, 20, 30, and 30 minutes, respectively. Finally, the samples were desiccated, mounted on aluminium stubs, coated with gold-palladium at a thickness of 200 Å, and examined by SEM (JEOL JEM 1600 Model).

### 2.14. Statistical Analysis

Results were expressed as mean ± standard deviation (SD). The intergroup variation was assessed by one way analysis of variance (ANOVA) followed by Post Hoc Tests Multiple comparison Bonferroni and statistical significance at *P* < 0.05 and *P* < 0.001 was calculated.

## 3. Results

### 3.1. *Giardia* Cycle

**Figure 1:**
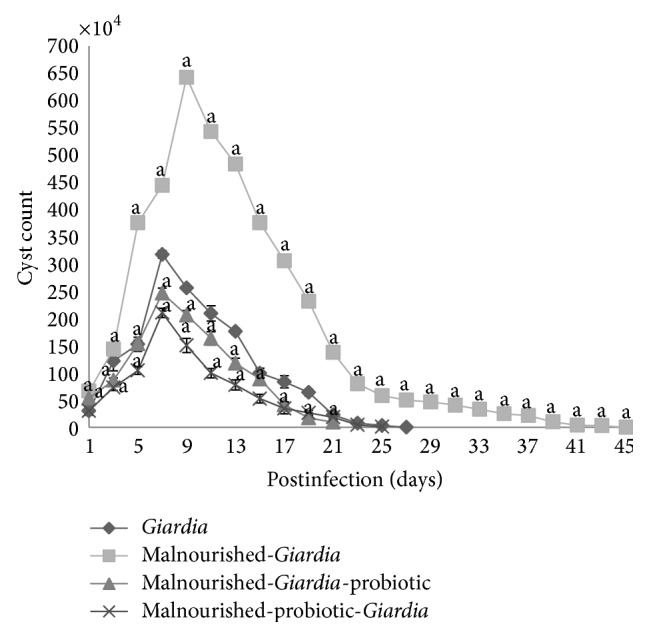
*Giardia* cysts count in faeces of different groups of mice on different days of postinfection. Values are mean ± SD, ^a^ *P* < 0.001 versus *Giardia*.

Malnourished mice challenged with *Giardia* trophozoites voided cysts gradually and had the highest cyst count on day 7 PI. Thereafter, they started receding and became *Giardia*-free by day 45 PI compared with day 29 PI in *Giardia-*infected mice. Instead, mice belonging to malnourished-*Giardia*-probiotic or malnourished-probiotic-*Giardia *groups had significantly (*P* < 0.001) lower cyst count and became *Giardia*-free by day 27 PI compared with *Giardia*-infected and malnourished-*Giardia*-infected mice (Figure 1). 

### 3.2. Intestinal Mass

**Figure 2:**
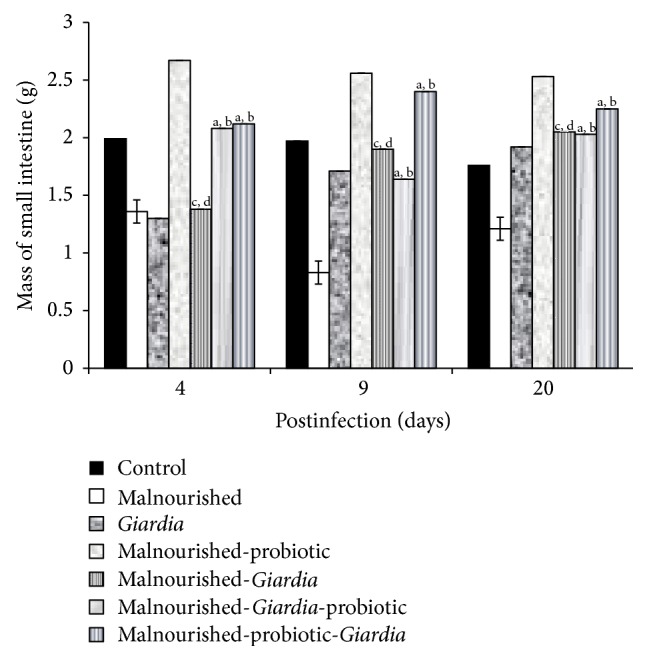
Mass of the small intestine of mice belonging to various groups. Values are mean ± SD; ^b^ *P* < 0.05 versus malnourished; ^a^ *P* < 0.05 versus *Giardia*; ^c^ *P* < 0.001 versus malnourished-probiotic; ^d^ *P* < 0.001 versus malnourished-probiotic-*Giardia*.

It was observed that mice belonging to malnourished-*Giardia*-probiotic or malnourished-probiotic-*Giardia *groups had significantly (*P* < 0.001) increased mass of the small intestine compared with malnourished and malnourished-*Giardia*-infected mice at each point of observation (Figure 2).

### 3.3. Intestinal Disaccharidases

**Figure 3:**
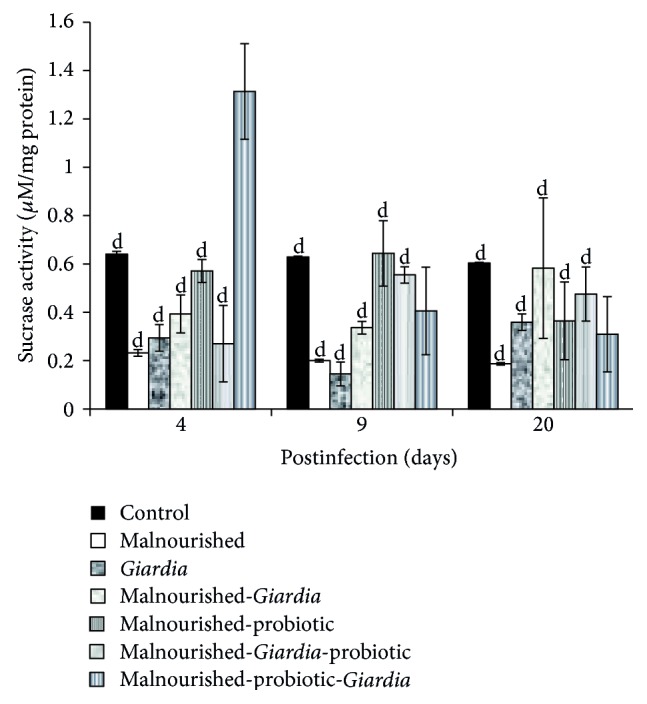
Sucrase activity (*μ*M/mg protein) in different groups of mice on different days of postinfection. Values are mean ± SD, ^d^ *P* < 0.05 versus malnourished-probiotic-*Giardia*.

**Figure 4:**
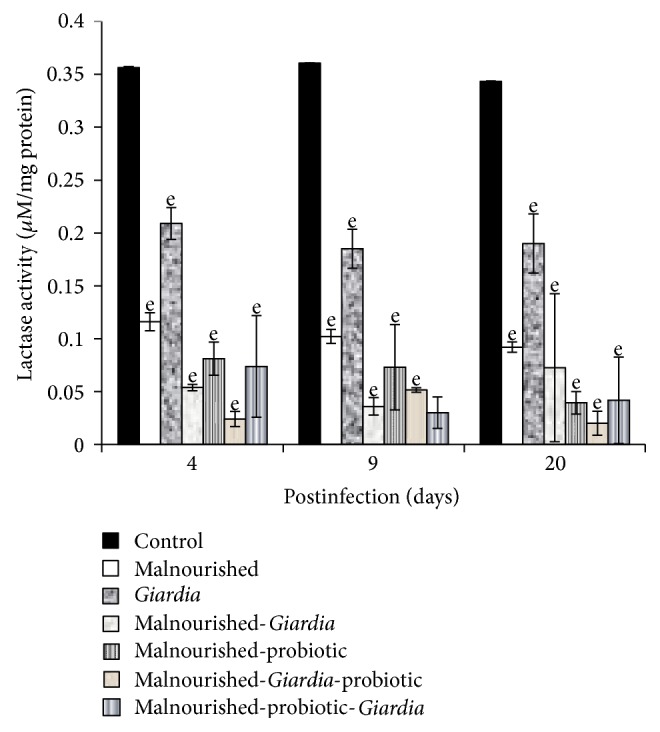
Lactase activity (*μ*M/mg protein) in different groups of mice on different days of postinfection. Values are mean ± SD, ^e^ *P* < 0.001 versus control.

**Figure 5:**
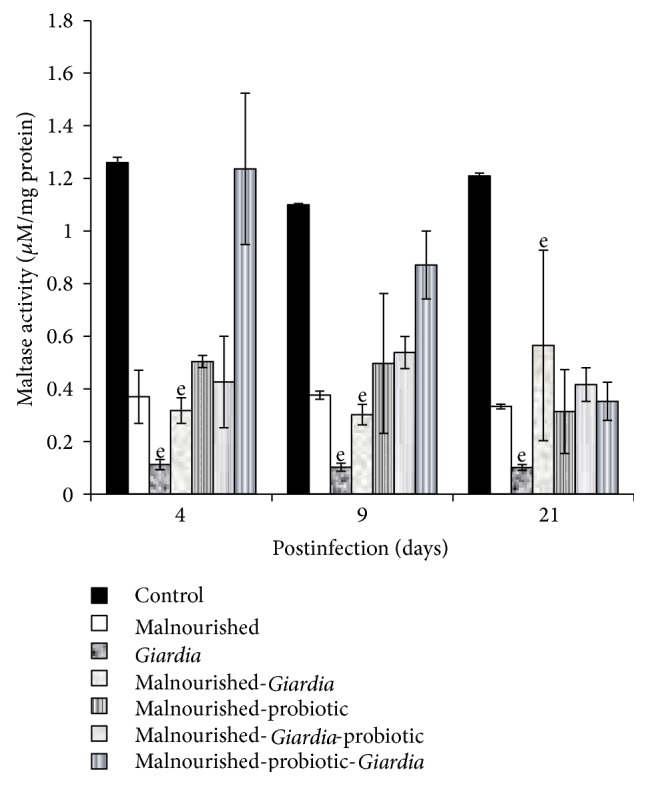
Maltase activity (*μ*M/mg protein) in different groups of mice on different days of postinfection. Values are mean ± SD, ^e^ *P* < 0.001 versus control.

 The activity of intestinal disaccharidases, mainly sucrase and lactase, in the BBM decreased significantly (*P* < 0.05) in mice belonging to all the groups compared with control mice at each point of observation, except in malnourished-probiotic-*Giardia* mice where sucrase activity was significantly higher in establishment phase (Figures 3 and 4). The maltase activity remained unaltered in malnourished and *Giardia-*infected mice at each point of observation but was significantly lower (*P* < 0.001) compared with control mice (Figure 5). It was interesting to note that the maltase activity increased in malnourished-probiotic-*Giardia* and malnourished-*Giardia-*probiotic mice at each point of observation compared with malnourished-*Giardia*-infected mice (Figure 5). 

### 3.4. Alkaline Phosphatase

**Figure 6:**
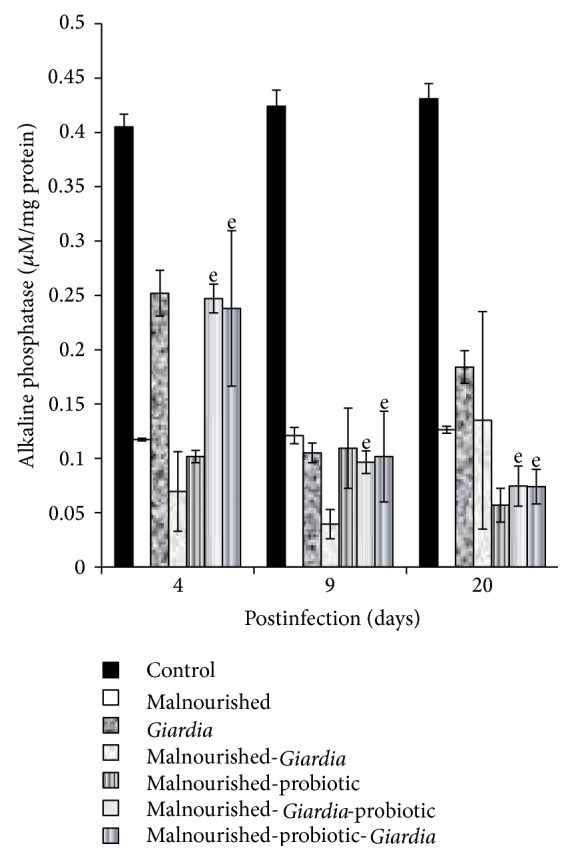
Alkaline phosphatase activity (*μ*M/mg protein) in different groups of mice on days 4, 9 and 20 postinfection. Values are mean ± SD, ^e^ *P* < 0.05 versus control.

The alkaline phosphatase activity decreased significantly (*P* < 0.05) in all the groups of mice compared with control at each point of observation (Figure 6). It was observed that *Giardia-*infected mice had more decreased alkaline phosphatase activity in both establishment and acute phase of infection than in decline phase. More specifically, the probiotic supplementation either prior to or simultaneously with *Giardia* infection to malnourished mice initially led to increased activity of alkaline phosphatase in the establishment phase but decreased in later phase of infection compared with malnourished, malnourished-*Giardia-*infected mice (Figure 6).

### 3.5. Scanning Electron Microscopy

**Figure 7:**
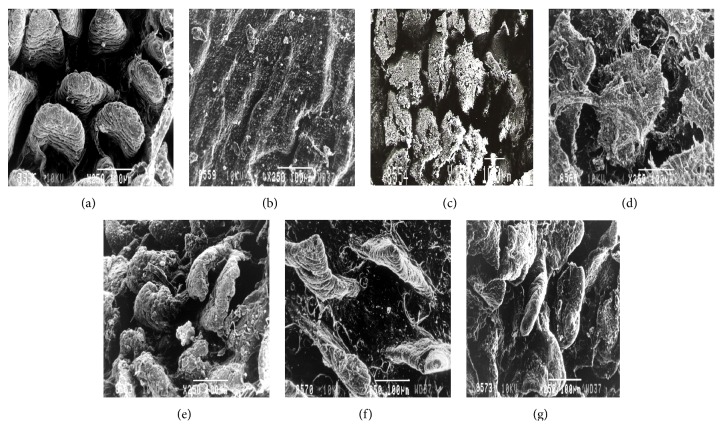
Scanning electron micrograph of the small intestine of mice belonging to different groups on day 9PI. (a) Control mice with well-organised and properly distributed microvilli (250x). (b) Malnourished mice showing mummified, disrupted, and blunted microvilli. Note the reduced length of microvilli (250x). (c) *Giardia-*infected mice showing damaged and disrupted microvilli along with deposition of surface exudates (150x). (d) Malnourished-*Giardia*-infected mice showing damaged, disrupted, and distant apart microvilli along with deposition of exudates (250x). (e) Malnourished-probiotic fed mice showing disorganized and shortened microvilli with better morphology (250x). (f) Malnourished-*Giardia-*probiotic mice showing altered crypt and villi morphology that are far apart from each other (250x). (g) Malnourished-probiotic-*Giardia-*infected mice showing somewhat preserved microvilli morphology and their orientation, along with the deposition of exudates (250x).

Scanning electron micrograph of the small intestine of control mice showed well-organized healthy microvilli (Figure 7(a)) compared with completely fused, mummified, and blunted microvilli in malnourished mice (Figure 7(b)). The small intestine of *Giardia-*infected mice had less-damaged disrupted microvilli along with deposition of exudates compared with severely disoriented, damaged microvilli along with deposition of exudates in malnourished-*Giardia-*infected mice (Figures 7(c) and 7(d)). However, nutritional manipulation of probiotic to malnourished mice showed somewhat restored microvilli morphology but their orientation and size were not as normal as that of control mice (Figure 7(e)). It was observed that probiotic supplementation either before or simultaneously with *Giardia* infection even to malnourished mice led to better morphology of the microvilli (Figures 7(f) and 7(g)) than malnourished, malnourished-*Giardia*-infected mice.

## 4. Discussion

Worldwide malnutrition and specific nutrient deficiencies are the leading cause of immunodeficiency, diseases, and death in developing countries [13, 23]. Therefore, the present study was designed to assess the underlying physiological protective mechanism of probiotic supplementation in malnourished-*Giardia-*infected mice.

 It was found that malnourished mice supplemented with probiotic either before or simultaneously with *Giardia*-infection had reduced percentage of cyst count and duration of infection than *Giardia *infected mice. The reduced severity of infection was due to the probiotic supplementation that is known to improve the anthropometric and biochemical parameters due to better colonization of healthy bacteria and corroborates with earlier studies [17–19, 24, 25]. The improved microbiota is essential for the maintenance and physiology of healthy bacteria as it synthesizes various substances (biotin, pantothenate, riboflavin, pyrodoxine, and vitamin K) important for metabolic processes and absorption of nutrients [17, 18, 26, 27]. This fact is supported by the present observation of increased mass of small intestine in probiotic-treated malnourished mice indicating healthy intestinal mucosa. 

The physiological activity of intestine was monitored by assaying the intestinal biomarkers mainly the intestinal disaccharidases (sucrase, lactase, and maltase) and alkaline phosphatase. Alkaline phosphatase, sucrase, and lactase are the villous tip enzyme marker while maltase is villous base marker suggesting that any alteration at these levels will directly affect the physiology of the small intestine which in turn affects the absorption of the nutrients and results into malnutrition. The decreased activity of mucosal disaccharidases and alkaline phosphatases in mice belonging to various malnourished groups concurs with earlier studies [28, 29]. The reduced disaccharidases activity in the present study is also paralleled with the severely diffused epithelial mucosal integrity of the small intestine as is evident by the present SEM observations. The observed structural alteration is in accordance with earlier studies [14, 30]. In these studies, scientists have also demonstrated the morphological and cellular alterations of microvillous membrane integrity in malnourished-*Giardia*-infected mice. Interestingly, the probiotic *L. casei* supplementation either before or simultaneously with *Giardia* infection to malnourished mice helped in restoring the intestinal mass, activity of the intestinal enzymes and is in accordance with the earlier observations [31, 32]. These scientists have also found the increased activity of brush border enzymes in rats treated with either sheep yoghurt containing *L. bulgaricus* and *Streptococcus thermophilus* or *L. johnsonii* La1.

In summary, findings from this study demonstrate that malnutrition makes the host more susceptible to *Giardia *infection due to altered gut morphology and intestinal dissacharidases deficiency which causes malabsorption and diarrhea. The possible mechanisms of probiotic therapy in malnourished-*Giardia-*infected mice may be due to normalisation of the gut microecology, intestinal permeability, and alleviation of intestinal brush border enzymes activity. However, the present observations need to be clinically correlated due to difference in the gut homeostasis of mouse and humans. 

## 5. Conclusion

Taken together, it can be concluded that both the atrophied gut and nutritional imbalance in malnourished hosts can be abrogated by the probiotic supplementation, used as an oral prophylactic adjuvant in renutrition diet. 

## Abbreviations

GRAS: Generally regarded as safe
MRS: De Mann Rogosa Sharpe
PBS: Phosphate-buffered saline
SEM: Scanning electron microscopy.
